# Professional nurses’ perspectives on the implementation of integrated chronic disease management model

**DOI:** 10.4102/curationis.v47i1.2585

**Published:** 2024-11-13

**Authors:** Avhaathu S. Dagume-Ndou, Thanyani G. Lumadi

**Affiliations:** 1 Department of Health Studies, Faculty of Human Sciences, University of South Africa, Pretoria, South Africa

**Keywords:** chronic diseases, implementation, integrated chronic disease management model, perspectives, professional nurses, South Africa

## Abstract

**Background:**

The integrated chronic disease management (ICDM) model is seen to be a promising strategy as it is gaining global acceptance as an approach to enhance healthcare delivery.

**Objectives:**

The study objectives were to explore and describe professional nurses’ perspectives on the model, identify challenges experienced during implementation and identify factors that could improve the model implementation.

**Method:**

An explorative, descriptive qualitative design was used, nine professional nurses from three selected facilities were interviewed. Data obtained from semi-structured individual interviews were analysed using thematic analysis and NVivo software.

**Results:**

Three main themes emerged: the professional nurses’ perceived benefits of the model, challenges experienced during implementation and recommendations to ensure effective implementation of the model. The perceived benefits of the model included the integration of care, adequate medication and the alleviation of stigma related to diseases. However, the challenges of the model included a shortage of staff, poor infrastructure and patients missing scheduled appointments. The study noted the need for adequate staff, infrastructure and ongoing training for the successful implementation of the model.

**Conclusion:**

Despite challenges in the South African healthcare system, professional nurses perceived the implementation of the model positively. The study highlighted the need for adequate staff, adequate infrastructure and ongoing training for staff.

**Contribution:**

This study outlined the challenges and recommendations identified, enabling healthcare organisations, policymakers and educators to develop targeted strategies for successful implementation. The findings will also inform future research focussed on optimising the utilisation of the model in the study setting and similar contexts.

## Introduction

The increasing prevalence of chronic diseases is posing a significant health problem globally, leading to higher rates of morbidity and premature death. The low- and middle-income countries (LMICs) are the most significantly affected, as 80% of deaths caused by chronic diseases occur in LMICs (Lebina 2021:13). South Africa, like many other countries around the world, is struggling with a growing burden of chronic diseases. In South Africa, 82% of consultations in primary healthcare (PHC) facilities are attributed to chronic diseases, with 65% of these patients having multiple chronic diseases. The substantial rise in chronic diseases in South Africa has posed significant challenges in the delivery of healthcare (Lebina 2021:23).

As a result, the South African National Department of Health (NDoH) has implemented the integrated chronic disease management (ICDM) model since 2011 to mitigate the impact of chronic diseases, promote the efficient use of primary healthcare services and improve health outcomes for its population (Godongwana, De Wet-Billings & Milovanovic 2021:2; Mahomed & Asmall 2017:2; Lebina 2021:7). The ICDM model is aimed at increasing facility competency and efficiency, patients’ better perception and engagement in the care of their chronic diseases, and a positive patient and healthcare professional experience. The model proposes reorganising facilities for better performance through measures, such as – improving bookings and patient flow, strengthening clinical management support by providing training and guidance on the delivery of the ICDM model, providing supervised self-management through participation, providing support at the community level, and prioritising health promotion and population screening to achieve the ICDM model’s outcomes (Godongwana et al. 2021:2; Lebina et al. 2020:3; Lebina 2021:15). The implementation of the ICDM model has garnered increasing attention and significance within healthcare settings. In South Africa, the ICDM model was initiated with the objective of addressing the burden of chronic diseases and promoting the efficient use of primary healthcare services (Mahomed & Asmall 2015:1; Lebina et al. 2019:2). The ICDM model was implemented at 42 primary healthcare facilities as a pilot study in three health districts: West-Rand in Gauteng, Bushbuchridge in Mpumalanga and DR Kenneth Kaunda in the North-West province.

Professional nurses are the core of the healthcare system and key stakeholders in the implementation of the ICDM model (Mahomed & Asmall 2017:2). Consequently, the successful implementation of the ICDM model is highly influenced by their perspectives. Their perspectives are of great value in comprehending the opportunities and challenges linked with the implementation of this model. The literature review revealed that previous studies have overlooked the qualitative perspective of professional nurses on the implementation of the ICDM model. This study was aimed at addressing this gap by exploring professional nurses’ perspectives on the implementation of the ICDM model, identifying challenges experienced by professional nurses when implementing the ICDM model, and identifying factors that can improve the implementation of the ICDM model at the primary healthcare facility of Mogale Local Municipality in Gauteng province, South Africa.

### Problem statement

The ICDM model is increasingly recognised as a feasible approach to effectively manage the complex needs of patients with chronic diseases in South Africa (Akugizibwe et al. 2023:2). The researcher identified that professional nurses’ perspectives can be utilised to strategise on how the implementation of the ICDM model can be improved. Given their vital role as implementers of the ICDM model at PHC facilities, motivated the need to explore professional nurses’ perspectives on the model, to understand challenges experienced during implementation and factors that can improve the implementation of the model at PHC facilities, particularly, in the Mogale Local Municipality of Gauteng province, South Africa. In addition, the researcher once visited two PHC facilities where the study was conducted and noted extended waiting times and overheard nursing staff expressing frustration about patients failing to attend their scheduled appointments and the shortage of staff.

## Research methods and design

This study employed an exploratory, descriptive qualitative design with the aim to explore and describe professional nurses’ perspectives on the implementation of the ICDM model in the selected public health facilities. The study was conducted during September and October 2022. Taherdoost (2022:54) affirmed that the main goal of qualitative research is to collect first-hand, detailed textual evidence and interpret it using particular interpretive methods.

The selected methodology is appropriate for the current study because the study aimed to explore and describe participants’ perspectives, and qualitative descriptive research was helpful because numeric data may not be enough to adequately convey how participants feel as perspectives cannot be quantified. In this study, professional nurses were offered an opportunity to articulate their perspectives on the implementation of the ICDM model at PHC facilities in Mogale Municipality of Gauteng province, South Africa.

### Research setting

The West-Rand District, located in the Gauteng province of South Africa, serves as the study setting for investigating professional nurses’ perspectives on the implementation of the ICDM model. Within the West-Rand District, the study specifically focussed on the Mogale Local Municipality, encompassing various healthcare facilities, including PHC centres. The settings were selected because Gauteng has the largest (14.7 million) percentage of South Africa’s population (White, Blaauw & Rispel 2020:5). West-Rand District was chosen as it was among the three public health districts where the ICDM model was piloted in 2011 (Mahomed & Asmall 2015:1). Accordingly, this setting provides a context for exploring the experiences and insights of professional nurses working in this specific geographic area. The study was conducted in three selected ICDM model-established PHC facilities that opened from Monday to Saturday and operated for 8–24 h each day in Mogale Local Municipality.

### Study population and sampling

The targeted population for this study consisted of all professional nurses implementing the ICDM model from the selected PHC facilities. The inclusion criteria for the sampling included – professional nurses who were permanently employed and held a degree or diploma. Additionally, having 1–10 years of experience in implementing the ICDM model, willing to participate in the research and having received training on the ICDM model were also decisive factors. The researcher was introduced to the professional nurses by the operational managers from all three PHC facilities. A non-probability purposive sampling procedure, as noted by Pitney and Packer (2020:166) was used to select nine professional nurses. Data saturation was attained at participant number eight, and one additional participant was added to see if any new information had appeared. No new theme emerged with participant number nine. Given that this study was exploratory, a sample size of nine participants was deemed appropriate.

### Data collection procedures

In this study, the researcher employed semi-structured individual interviews and an interview guide comprised of a question requesting demographic details. An introductory question was asked: ‘What is your understanding of the ICDM model?’ Open-ended questions were asked based on one outcome of the ICDM model including benefits, acceptability of the model to staff, number and availability of staff and equipment, cleanliness of the facility, waiting time, adherence to guidelines, challenges experienced and strategies to improve the implementation of the model. The interview guide was pre-tested with three participants within the identified study area. Participants who participated in pre-testing of the interview guide were excluded from the main study. The interview guide was revised to incorporate age and gender as demographic factors and an additional question on the perceived benefits of implementing the ICDM model was added.

The researcher visited each PHC facility after receiving approval from the West-Rand District Family Medicine to submit written permission letters to carry out the study. The researcher comprehensively informed the operational managers of the research methodology and they gave verbal permission to collect data. The researcher sought the help of operational managers to find participants who matched the study’s criteria, and the operational managers provided the names of professional nurses who satisfied the study’s criteria. Subsequently, depending on their willingness to engage in the study, a sample of professional nurses was selected. All participants in the study who met the study criteria were told what the study’s purpose was. The researcher then obtained written informed consent and scheduled interviews based on the participants’ availability.

The researcher conducted interviews for approximately 30 min with the professional nurses in their field of work, in a private room provided by the PHC facility. The researcher also took field notes during the interview to identify possibly contradictory statements and follow up on new, interesting topic areas not addressed in the interview guide. Individual interviews were audio recorded with the consent of the participants.

### Data analysis

The researcher sought the help of an independent coder to analyse the audio-recorded data. The NVivo software was utilised to analyse the semi-structured data. The steps followed for the analysis of the data in this study included the verbatim transcription of the raw data, viewing it and thoroughly studying the transcripts to have an in-depth understanding of the data while determining their significance. Subsequently, the data were prepared for coding, and the linked concepts and categories were correctly developed and combined into themes for validity and clarity. The data were reviewed and discussed by the researcher and the independent coder to confirm and ensure the representation of the range of views expressed by participants.

### Trustworthiness

Cited in Brink et al. (2018:157), Lincoln and Guba’s (1985) rigour and trustworthiness criteria were used in this study; hence, each of the following factors was followed to assure trustworthiness: credibility, transferability, dependability and confirmability. The audio-recorded interviews and verbatim transcription endorsed the credibility of the data. For data transferability, the researcher thoroughly described the context of the study, including an accurate description of the participants, the sampling process, and the date, time and location of data collection. Dependability was attained by the description of the data collection methodology, as well as the subsequent processes employed for analysing the data and interpretation. Furthermore, the researcher conducted member checks, allowing participants to review and validate the researcher’s interpretation of their responses, thus ensuring accuracy. An independent coder and direct quotes from the participants in the study report were used to ensure confirmability.

### Ethical considerations

The study received ethical clearance from the University of South Africa College of Human Sciences Research Ethics Committee (64045919_CREC_CHS_2022). Approval to conduct the study was granted by the Gauteng Department of Health (NHRD) (GP_202208_067) as well as verbal consent from facility operational managers. All data were obtained independently. The researcher obtained signed informed consent to participate from the participants after a comprehensive overview of the research process was explained.

The ethical principles of justice, privacy, confidentiality and anonymity were maintained. All eligible participants were given equal opportunity to engage in the study. There was no identifiable data used for participants or facilities. The researcher employed numbers and alphabets for participants and facilities. Participation in the study was voluntary, and professional nurses were given the freedom to withdraw from the study at any time without consequences. Each PHC facility provided a private room where interviews were conducted. The researcher maintained confidentiality by not connecting identifiable information to the raw data, and data were password-protected and secured on encrypted devices. There was no identifiable data used for participants or facilities. The researcher employed numbers and alphabets for participants and facilities to help maintain anonymity.

## Results

### Demographic characteristics of participants

The study included professional nurses employed at three selected PHC facilities of Mogale Local Municipality in Gauteng province. These professional nurses have been using the ICDM model for 2–10 years. The participants were eight females and one male. The age range of the participants varied between 41 and 60 years. All participants indicated that they had received training in Nurse-Initiated Management of Anti-Retroviral Therapy (NIMART), Primary Care 101 (PC 101) and HIV Counselling and Testing (HCT). Three participants received training in PHC; however, two participants had attended the Practical Approach to Lung Health in South Africa (PALSA) programme as shown in Table 1.

**TABLE 1:** Demographic characteristics of participants.

Participant number	Age (years)	Gender	Number of years using the ICDM model	Training attended	Clinic
Participant 1	52	Female	7	NIMART /PC101	A
Participant 2	57	Female	10	PHC/PALSA/NIMART/PC101	A
Participant 3	50	Female	2	PC101/NIMART	A
Participant 4	50	Female	5	NIMART /PC101	B
Participant 5	53	Female	7	PHC/PALSA/NIMART/PC101	B
Participant 6	52	Female	9	NIMART /PC101	C
Participant 7	49	Male	5	PHC/NIMART/PC101	C
Participant 8	55	Female	5	NIMART /PC101	B
Participant 9	54	Female	4	NIMART /PC101	C

*Source*: Dagume-Ndou, A.S., 2023, *Professional nurses’ perspectives on the implementation of the integrated chronic disease management model in Mogale Local Municipality, Gauteng Province, South Africa*, MPH dissertation, University of South Africa, p. 62, viewed from https://uir.unisa.ac.za/handle/10500/31356

ICDM, integrated chronic disease management.

### Main themes and sub-themes

The main themes and sub-themes are presented in Table 2.

**TABLE 2:** An outline of the identified themes and sub-themes.

Themes	Sub-themes
1. Professional nurses’ perceived benefits	1.1 Integration of care 1.2 Adequate medication supply 1.3 Alleviation of stigma relating to diseases
2. Challenges experienced by professional nurses during the implementation of the ICDM model	2.1 Shortage of staff 2.2 Poor infrastructure 2.3 Patients missing scheduled appointments
3. Professional nurses’ recommendations to ensure effective implementation of the ICDM model	3.1 Adequate staff 3.2 Adequate infrastructure 3.3 Provision of training for staff

*Source*: Dagume-Ndou, A.S., 2023, *Professional nurses’ perspectives on the implementation of the integrated chronic disease management model in Mogale Local Municipality, Gauteng Province, South Africa*, MPH dissertation, University of South Africa, p. 63, viewed from https://uir.unisa.ac.za/handle/10500/31356?show=full

ICDM, integrated chronic disease management.

#### Theme 1: Professional nurses’ perceived benefits

Theme 1 outlines professional nurses’ perceived benefits of the ICDM model implementation.

**Sub-theme 1.1: Integration of care:** The primary objective of the ICDM model is to provide comprehensive and coordinated treatment and care to patients with chronic diseases (Godongwana et al. 2021:8). Most participants indicated that their understanding of the model is that it entails an integration of chronic disease management, which leads to improved patient care outcomes. Participants indicated that the ICDM model facilitates close collaboration and effective information sharing among diverse healthcare providers – physicians, nurses, specialists, social workers and others. In addition, this coordination facilitates the provision of consistent and integrated care to patients across various healthcare settings and disciplines:

‘Okay, my understanding is that you integrate the services. ICDM is about the integration of chronic disease management. So, the healthcare professionals work together to provide services needed for chronic disease patients so that we can be able to render quality care to these patients.’ (P1, Clinic A, 52 years old, Female)

‘The ICDM model is really helpful for us as healthcare professionals because it allows us to easily share information and responsibility for the well-being of our patients.’ (P2, Clinic A, 57 years old, Female)

Participants stated that by using the model they can deliver comprehensive care for various chronic diseases, such as HIV, hypertension and diabetes; hence, the potential benefit of providing comprehensive care to patients with comorbidities in one visit. Simultaneous treatment and care of chronic conditions during a single clinic visit offer a comprehensive and continuous method of addressing comorbidities, thus providing tailored treatment that addresses the specific needs of the patients.

In their account, with this model, when a patient with multiple chronic diseases seeks healthcare services, the patient is helped in one room. This arrangement facilitates early detection and management of any potential health issues, thereby reducing the risk of complications:

‘In the ICDM model, everything is integrated in one. You are able to identify and address health issues early on, and if necessary, refer a patient to get further help. This can potentially prevent more serious complications.’ (P3, Clinic A, 50 years old, Female)

‘The ICDM model is all in one place … but the good thing is that you can manage one patient and pick-up the problems that arise. Maybe you are dealing with HIV patient, who have developed diabetes in the process, now you know exactly how to treat the patient.’ (P4, Clinic B, 50 years old, Female)

‘The ICDM model speaks of integrating all the diseases. We are looking at all levels of how the patient is coming to the clinic, and the different solutions that they want. If a patient comes to you with chronic diseases and has minor sicknesses, it means the patient cannot run around and go to another side of the clinics. If the patient has come and she is sick or he sick, we’re going to sit the patient in one room and attend to all her needs.’ (P6, Clinic C, 52 years old, Female)

The findings of the study show that the ICDM model offers a comprehensive approach to delivering integrated care. The findings of this study are comparable with the study by Mwagomba et al. (2018:28), which highlighted that the ICDM model integrates all chronic diseases in contrast to a particular dealing with them, either as HIV or NCD. The study findings demonstrated that PHC facilities are effectively delivering integrated care services, hence ensuring the provision of continuous care for individuals with chronic diseases.

**Sub-theme 1.2: Adequate medication supply:** The principle of the ICDM model further includes the provision of sufficient quantities of medications and other supplies (Lebina et al. 2019:2). Adequate medication supply is essential for continuous patient care. Most of the participants expressed contentment with the handling of essential medication. Participants mentioned the following:

‘There is an adequate supply of medication. Medication supply is timely. Every Monday, we conduct a stock check to ensure that any items that are in short supply are ordered on time.’ (P6, Clinic C, 52 years old, Female)

‘We don’t have any problem with the availability of medication. The medication is always available.’ (P8, Clinic B, 55 years old, Female)

‘The CCMDD program makes sure that the patients receive their medication easily and conveniently.’ (P1, Clinic A, 52 years old, Female)

‘CCMDD reduced the strain in our healthcare facility and enhanced patient adherence.’ (P2, Clinic A, 57 years old, Female)

‘CCMDD has improved the management of chronic diseases patients.’ (P7, Clinic C, 49 years old, Male)

Participants indicated that the Central Chronic Medicines Dispensing and Distribution (CCMDD) programme was offered at three PHC facilities; despite that, there was timely delivery and sufficient availability of medication in all PHC facilities. The findings of this study are inconsistent with previous studies on the implementation of the ICDM model, which indicated challenges such as delayed medication delivery and medication stock-outs (Godongwana et al. 2021:6; Lebina et al. 2020:10; Malebo 2022:44). Adequate medication supply positively impacts patient outcomes by preventing worsening health conditions and mortality. The findings of the study indicated positive outcomes from adhering to medication management guidelines in the implementation of the ICDM model. This approach improves operational efficiency by allowing patients to receive their medication on the same day, thereby reducing the need for them to return on a different date as well as decreasing the possible risk of patients defaulting on their treatment.

**Sub-theme 1.3: Alleviation of stigma relating to diseases:** Participants highlighted that the implementation of the ICDM model has been found to effectively reduce the discrimination and stigmatisation experienced by patients with chronic diseases. Most participants endorsed the ICDM model as a promising initiative and expressed that the ICDM model reduced the separation of patients, as all patients with different chronic diseases are together in the same waiting area. For example, there is no separation of patients who are HIV-positive and patients with other chronic diseases. The following affirm participants’ perspectives:

‘But it is a nice thing they did because it removed even the stigma attached to some diseases. Because now patients can sit all together and discuss and feel free. So, it does relieve some burden in terms of stigma.’ (P1, Clinic A, 52 years old, Female)

‘… the ICDM model I think it’s more of taking care of the chronic patient in the same setting so that it makes it better so that patients don’t feel like they’re being isolated from other patients; for instance, HIV patients usually defaulted due to concerns about their status being known by others.’ (P4, Clinic B, 50 years old, Female)

‘With the ICDM model, patients are not separated according to their sickness, all patients share the same waiting room and collect medication from the same place with other chronic disease patients which is good because in the past HIV patients were treated at their special clinic and it was easy to identify patients who were HIV-positive.’ (P7, Clinic C, 49 years old, Male)

One of the benefits associated with the implementation of the ICDM model is its ability to promote the integration and non-segregation of patients. In all the PHC facilities, participants reported that the implementation of the ICDM model significantly contributed to a reduction in HIV-related stigma because of the lack of segregation of patients receiving care for chronic diseases. Participants explained that previous practices of separating patients based on their illness facilitated the identification of patients receiving treatment for diseases like HIV/AIDS in PHC facilities. HIV patients used to default on their treatment because they had their own designated settings.

The findings of content analysis conducted in the rural Agincourt sub-district of Mpumalanga province, South Africa are comparable to the findings of this study, in that, the implementation of the ICDM model was perceived to have played a role in reducing HIV-related stigma in PHC facilities (Ameh et al. 2020:2). The findings of this study demonstrate that the implementation of the ICDM model produced the expected outcome of reducing HIV-related stigma. However, the professional nurses indicated that there are challenges experienced when implementing the ICDM model.

#### Theme 2: Challenges experienced by professional nurses during the implementation of the integrated chronic disease management model

Theme 2 outlines the challenges experienced by professional nurses during the implementation of the ICDM model. This section discusses the sub-themes presented in Table 2.

**Sub-theme 2.1: Shortage of staff:** The participants identified the shortage of staff as one of the main challenges encountered during the implementation of the ICDM model, within their respective facilities. Participants indicated that there is no substitution or temporary staff hired when a staff member is ill or on leave; thus, the remaining staff must manage the same number of patients. One of the participants made reference to the resignation of a staff member, which has resulted in an ongoing vacancy for that position. This situation has created a burden for the participants as it impacts service provision in accordance with the guidelines. Participants highlighted the issue of an unbalanced patient-to-nurse ratio, which creates strain on healthcare providers. Participants noted the following:

‘Yes, staff availability is the problem here because we have many patients in this clinic, mostly chronic patients. Yes, but limited numbers of staff. I will say somehow, we lack because of the patient ratio, because there are more chronic patients, and then the sisters, we are limited.’ (P1, Clinic A, 52 years old, Female)

Similarly, other participants complained that nurses are faced with the challenge of managing an overburdened healthcare system, characterised by a high patient volume and a scarcity of human resources:

‘I mean, because the program is always overbooked because unfortunately, that is where we are as a facility. Let’s say, you book 120 patients for that specific day. If an additional 20 patients arrive without scheduled appointments, the total number of patients would be 140. Considering the limited capacity of four staff to manage this number of patients, the situation is undeniably challenging. I mean, this is absolutely ridiculous …’ (P2, Clinic A, 57 years old, Female)

‘ICDM is a good thing but … it needs staff … staff is a problem. Our facility is usually filled with chronic patients. Sometimes, there isn’t enough time to spend with one patient and you are forced to rush to the next patient on the line.’ (P3, Clinic A, 50 years old, Female)

‘Our patients are many in number, but the staff is very few, others resign and others you find that at one time when it’s time for the consultations, the patients will be more because some nurses will be sick and because of the shortage of staff, then it will not be a good day for me …’ (P6, Clinic C, 52 years old, Female)

‘The staff is limited, and there is a vacant position in our facility due to the resignation of a former staff member. This situation has placed a lot of pressure on us, as we are still required to attend to the same or an increased number of patients.’ (P7, Clinic C, 49 years old, Male)

‘The number of staff, I can say … so due to the condition that we are now, we are short staff, yes, we have got limited staff.’ (P8, Clinic B, 55 years old, Female)

In all PHC facilities, participants expressed concerns about insufficient staff. Participants reported experiencing a high level of distress because of the consistent influx of patients; they expressed concerns that limited time is spent with patients which is primarily attributed to their heavy workload because they are required to attend to a significant number of patients on a daily basis.

The findings of this study are consistent with a cross-sectional study conducted in South Africa. This previous study examined 16 PHC clinics in two health districts and found that a lack of human resources may have contributed to a lower commitment to implementing the ICDM model (Lebina et al. 2019:11). The study findings indicated that the shortage of staff negatively impacts the quality of patient care.

**Sub-theme 2.2: Poor infrastructure:** Most of the professional nurses expressed concerns over poor infrastructure. The participants complained that the implementation of streams of care is challenging because of the limitations posed by the infrastructure of the PHC facilities. Concerns were voiced about the inadequate size of the consultation spaces; this limits a comprehensive assessment of the patients and subsequently affects the quality of patient care. These participating nurses indicated that the facility has limited space, also resulting in patients being required to wait outside, sometimes in adverse weather conditions. Participants expressed the need for the Department of Health to address infrastructure issues and emphasised the urgency of developing facility infrastructure that adheres to the principles of the ICDM model and facilitates collaboration and patient-centred care. They emphasised that such an infrastructure would greatly improve the quality of care for patients with chronic diseases. Participants stated:

‘If consulting rooms are few, how are you going to manage? Department needs to do something with the structure. The number of consultation rooms is limited, and their size is small. In addition, the waiting area is also small.’ (P1, Clinic A, 52 years old, Female)

‘The rooms are very small, making it difficult to do a thorough assessment. So, proper infrastructure is necessary.’ (P5, Clinic B, 53 years old, Female)

‘Because we are the only government facility that caters to a population of plus or minus 45 000 and in our facility; we only have eight consulting rooms. Limited space capacity is currently a major concern.’ (P7, Clinic C, 49 years old, Male)

In all the PHC facilities, participants reported challenges in implementing the ICDM model guidelines, for the three distinct streams of care – chronic care, minor ailments care and maternal and prenatal care – because of the existing limited clinic infrastructure. The findings of the study align with findings reported by Malebo (2022:35) – that adherence to the ICDM model guidelines for the three streams of care was compromised because of poor infrastructure. The current study’s findings also demonstrate that inadequate infrastructure hinders patients’ access to comprehensive and integrated care, thus affecting patients’ health outcomes.

**Sub-theme 2.3: Patients missing scheduled appointments:** The responses also highlighted that patients’ poor adherence to scheduled appointments was a significant barrier to implementing the ICDM approach. Some of the participants expressed their frustration regarding this issue, complaining that chronic patients are compromising the effectiveness of the ICDM model because of their non-compliance with the booking system and frequent defaults.

Participants described how patients often miss appointments because they have to report to work on the scheduled checkup date; this is despite the attempts the nurses make to educate patients on the need to stick to their appointments. The participants also made reference to employer’s need to care and be considerate towards employees who have chronic illnesses:

‘We schedule a specific number of patients each day, but some of them do not attend their scheduled appointments. Instead, they arrive on a different date to the one they were originally booked for. As a result, the total number of patients increases, leading to a high patient volume on that particular day.’ (P1, Clinic A, 52 years old, Female)

‘A lot of our frustrations come from patients who default on their appointment dates and as much as you educate them it makes no difference, I think the patients have got this thing that they can come to the clinic whenever it’s suitable to them … On numerous occasions, patients have expressed their inability to visit the facility due to work commitments, because of employer refusal to grant them time off to attend their scheduled appointments. I am concerned about this issue because employees have a significant responsibility towards their workers, particularly in matters related to health … It is not acceptable to me.’ (P2, Clinic A, 57 years old, Female)

‘I think it’s the defaulters, they don’t come on the given dates or sometimes they are taking a six-month treatment, and they don’t come back after six months. Then you find that when they come, their blood pressure and viral loads are high because they last took treatment six months ago.’ (P5, Clinic B, 53 years old, Female)

‘Our big problem is the defaulting on the different programs that have been implemented, like the collection from CCMDD but the people are defaulting even in other ways; people always miss their appointments.’ (P6, Clinic C, 52 years old, Female)

Participants indicated that the high patient volume can be attributed to patients failing to attend their scheduled appointments. All the participants reported that a significant number of chronic patients failed to adhere to their scheduled appointments; patient’s non-adherence to scheduled appointments negatively impacts their care, leading to deterioration in their health condition. This finding supports a study by Malebo (2022:22), that claimed that patients who miss their scheduled appointments default on their medical treatment. Poor attendance of scheduled appointments has a negative impact on clinics’ adherence to proposed ICDM guideline activities. Regardless of the challenges experienced, professional nurses alluded to the fact that these recommendations might help in the management of the ICDM model as explained in ‘Theme 3’.

#### Theme 3: Professional nurses’ recommendations to ensure effective implementation of the integrated chronic disease management model

Theme 3 provides professional nurses’ recommendations for efficient ICDM model implementation. This section discusses the sub-themes presented in Table 2.

**Sub-theme 3.1: Adequate staff:** Most of the participants mentioned that the ICDM model implementation requires more staff than is currently available. Participants indicated that the provision of adequate staff should be in line with the goals and objectives of the ICDM model. They articulated that increasing the number of staff members would enable them to provide high-quality care and prioritise patient well-being over the need to reduce and expedite the movement of the queues. The perspectives of the individuals are presented in the following comments:

‘I personally think that the ICDM is a good model, but it definitely requires more staff to be effective. That’s why I’m suggesting that perhaps having enough staff would allow us to focus more on taking care of the patient, rather than being overwhelmed with pushing through queues and worrying about statistics. Yes, it’s important to make sure you’re getting good quality care. It’s not just about the stats, but ensuring good quality for our patients.’ (P4, Clinic B, 50 years old, Female)

‘If we have more staff, even the patients would be happier because some of them complain about having to wait for a long time. Government needs to attend to the shortage of staff.’ (P7, Clinic C, 49 years old, Male)

‘As I said, the influx of chronic care patients is a problem, while having insufficient human resources to meet this demand. You know, and then the staff issue also comes in because the more patients you have, the longer the waiting period is going to be. So, the provision of sufficient staff is an urgent issue.’ (P9, Clinic C, 54 years old, Female)

Participants identified staff shortage as one major limiting factor in implementing the recommended ICDM model’s guidelines. Some participants attributed long waiting times and the influx of chronic patients to the shortage of staff. Participants emphasised the importance of adequate staffing for an effective implementation of the ICDM model. This study’s findings align with the findings of Lebina et al. (2020:8), which suggest that ensuring enough staff members, on a rotational basis, can enhance the implementation of the ICDM model.

**Sub-theme 3.2: Adequate infrastructure:** The planning and development of adequate infrastructure, space and facility design were recognised by participants as essential prerequisites for the successful implementation of the recommended ICDM model’s activities (as represented in the remarks that follow):

‘I think the first thing they should do is try to increase the infrastructure before implementing any changes. I know the department always claims they don’t have enough money, but if they can find a way to accommodate the number of patients or the population the clinic serves, it would be much easier. For example, here at the COVID side, we only have three rooms. In the past, we used to see around 150 or more patients per day. But if we had a bigger clinic with more rooms and staff, it would be much easier for everyone.’ (P7, Clinic C, 49 years old, Male)

The findings of the study suggest that adequate infrastructure and space are essential prerequisites for achieving the desired outcomes of the ICDM model.

**Sub-theme 3.3: Provision of training for staff:** Participants identified adequate staff training on the principles of the ICDM model as a significant component for the adoption and sustainability of high adherence to the model; this training should be made available to everyone, rather than just a few selected people. The perspectives of the participants are expressed in the comments that follow:

‘Staff need more training on how to integrate the services. I think they should definitely consider in-service training for all staff.’ (P1, Clinic A, 52 years old, Female)

‘It’s important to have ongoing training and workshops for the staff. That way, they’ll know exactly what to do and really understand their roles. I think it would be a good idea for all staff to go for some additional training.’ (P4, Clinic B, 50 years old, Female)

‘I really appreciate that we have training sessions to attend. However, the ICDM model champion is the one who usually goes most of the time. I strongly believe that the addition of service training is necessary in order to improve the efficacy of the ICDM model’s implementation.’ (P7, Clinic C, 49 years old, Male)

In all PHC facilities, participants expressed significant concern regarding insufficient training in implementing the ICDM model. Participants indicated that only a selected few staff members, mainly clinical managers and the ICDM model champions attended the training. These findings are consistent with those from a previous study conducted in Bushbuckridge that examined the perceptions and experiences of clinic managers on the implementation of the ICDM model in PHC clinics; the results showed that the workshops were primarily attended by clinic managers (Malebo 2022:41).

The findings of the current study, therefore, suggest that regular training and workshops are essential for staff members to acquire knowledge and a thorough understanding of their tasks and responsibilities. Furthermore, it is recommended that all staff members receive in-service training, and not just the model champions.

## Discussion

The study objectives were to explore and describe professional nurses’ perspectives on the implementation of the ICDM model, identify challenges experienced by professional nurses when implementing the ICDM model, and identify factors that can improve the implementation of the ICDM at a PHC facility in Mogale Local Municipality of Gauteng Province, South Africa. In this study, professional nurses’ perspectives on the ICDM model implementation were influenced by a wide variety of issues – perceived benefits, challenges experienced and recommendations to ensure the model’s effective implementation and improvement.

Professional nurses expressed positive perspectives on the implementation of the ICDM model. Positive perceptions, experiences and attitudes towards the implementation of the ICDM model have also been expressed by previous studies (Lebina 2021:31; Mahomed & Asmall 2017:3). Professional nurses emphasised that the ICDM model has proven to be effective in delivering integrated care, ensuring adequate medication supply and reducing stigma associated with certain diseases. The findings of the study revealed that the ICDM model helps to effectively integrate care for patients with chronic diseases by all three selected PHC institutions; this aligns with other studies (Mwagomba et al. 2018:28). Professional nurses reported that simultaneously addressing all aspects of patient’s health resulted in enhanced management of chronic diseases. Findings from this study highlighted that the ICDM model promotes collaboration and facilitates the sharing of information and responsibility among healthcare providers for the well-being of patients. Professional nurses affirmed that collaboration among healthcare providers enhances the quality of healthcare services in PHC facilities (Nene 2024:6).

The findings of the study also ensure the provision of adequate medication supply in all PHC facilities. Professional nurses noted that the use of CCMDD not only increased the availability of medication but also improved the standard of treatment for people suffering from chronic diseases given that it enhanced patient adherence as it ensures a timely supply of medication to patients. These findings concur with previous studies on the benefits of implementation of the ICDM model, which had identified one of the challenges in healthcare facilities as delayed medication delivery and medication stock-outs (Godongwana et al. 2021:6; Lebina et al. 2020:10; Malebo 2022:44). The study suggested that the implementation of the ICDM model alleviated stigma, particularly HIV-related one. The findings of the Agincourt study conducted in Mpumalanga province, South Africa are comparable with the findings of this study, as both studies indicate that the implementation of the ICDM model contributed to a reduction in HIV-related stigma in PHC facilities (Ameh et al. 2020:2). However, professional nurses have reported challenges in implementing the ICDM model.

Professional nurses mentioned challenges such as limited resources, including staff shortages. The study revealed that the shortage of staff has a negative impact on the provision of services, as outlined by the ICDM model guidelines. The shortage of staff is a major human resources challenge faced by many low- and middle-income countries, including South Africa, when implementing health measures (Malebo 2022:43). In all the PHC facilities, professional nurses attributed long waiting times and the influx of chronic patients to shortage of staff. The findings of the study are comparable with those of a study carried out by Ameh et al. (2020:8), which identified staff shortage as a primary challenge in providing quality care in PHC facilities. Professional nurses acknowledged that they often have to spend limited time with patients because of a shortage of staff. Professional nurses also expressed frustration with the lack of replacement when a staff member is on leave or resigns, resulting in having to attend to the same or even more patients. Mahomed and Asmall (2017:1) voiced the same views and noted that despite PHC facilities’ readiness and the commencement of appropriate management, the lack of necessary resources, including the shortage of staff at the PHC facilities, provides a major impediment to the successful implementation of the ICDM model.

An additional significant factor negatively affecting the implementation of the ICDM model was attributed to poor infrastructure. The professional nurses raised concerns over poor infrastructure, such as the working spaces being too small and there being a limited number of consultation rooms. Professional nurses identified that poor infrastructure creates a challenge in delivering the comprehensive treatment envisioned by the ICDM model. The findings of this study are comparable with the findings presented by Lebina et al. (2020:8), who noted that the current infrastructure of PHC facilities is limited in size and has a limited number of consulting rooms. Chronic patients missing scheduled appointments was also discovered to be compromising the implementation of the ICDM model. The common issue of patients constantly missing scheduled appointments confirms both the challenges and effects of appointments’ non-adherence by patients, in the context of the ICDM model. The findings of the study are comparable with the findings reported by Malebo (2020:43), who asserted that patients with chronic diseases often fail to attend their scheduled appointments at PHC facilities or decentralised pick-up stations in South Africa. Furthermore, the findings of the study indicated that patient’s non-adherence to scheduled appointments results in PHC facility’s non-compliance with the recommended guidelines which negatively impacts the provision of health services at the facilities. Regardless of the challenges experienced, professional nurses identified the following contextual factors that could facilitate the implementation of the ICDM model.

The identified contextual factors for effective implementation of the ICDM model were the provision of adequate staff, infrastructure and training. The study discovered that both staff and infrastructure factors play a key role in the successful implementation of the ICDM model. Professional nurses highlighted the importance of maintaining adequate staffing levels in healthcare facilities for effective implementation of the ICDM model (Lebina et al. 2020:8). Professional nurses expressed that improving the PHC facilities infrastructure through expanding the space for chronic disease patients, providing more specialised rooms for chronic patient management, and maintaining safe and clean environments for patients and staff would ultimately result in improved management of chronic diseases. Professional nurses have expressed concerns about feeling excluded, as only the ICDM champions are given the opportunity to attend most of the training courses. They have also highlighted the lack of ongoing training opportunities, which has limited their capacity to effectively employ the components of the models (Malebo 2022:41). Professional nurses claimed that training is imperative for all healthcare personnel to gain insight into the principles, as well as the processes of the ICDM model. Professional nurses indicated that adequate staff training is a significant component in promoting the adoption and sustainability of high adherence to the model. The findings suggested that there should be additional training and that this training should be made available to everyone, rather than just a few selected staff members.

The findings of the current study indicated that addressing these aforementioned factors will provide an optimal context for the successful implementation of the ICDM model. This will ultimately lead to improved patient care outcomes and increased efficiency in the provision of health services. These findings are consistent with a cross-sectional mixed-method study conducted in two health districts in South Africa, which also emphasised the significance of adequate staff, infrastructure and training in facilitating the implementation of the model (Lebina et al. 2020:10).

### Strength and limitations

The study offered a more detailed overview of how professional nurses view the implementation of the ICDM model. Additionally, the participants were free to describe and evaluate their experiences as well as offer suggestions for improvement because of the use of an explorative design. Similarly, using purposive sampling enabled the researcher to choose the sample based on participants’ knowledge of the phenomenon under study, helping in the collection of detailed data. This study only included professional nurses with 1–10 years of ICDM model experience; hence, other professional nurses with less experience were excluded from the study. In addition, the study included only participants who voluntarily agreed to participate, while those who did not provide consent were excluded. The perspectives of professional nurses who agreed to participate in the interviews may have been similar or different from those who declined to be interviewed or those who did not meet the specific criteria for inclusion. The findings, as a result, cannot be generalised to the entire population of professional nurses in the country.

The study sample size was fairly small, which limits the generalisability of its findings to the wider population of professional nurses implementing the ICDM model. To address this limitation, the researcher employed a meticulous study design, ensured transparency in reporting and provided a thoughtful unbiased interpretation of the findings.

This study was conducted exclusively in the Mogale Local Municipality of Gauteng province, South Africa, while other local municipalities that utilised the ICDM model were not part of the study. In addition, the study focussed on three PHC facilities which was a fairly small number; hence, the results are limited to the specific PHC facilities where the study took place. The small sample size, consisting of only three PHC facilities, means that the results cannot be generalised to other settings.

### Recommendations

The following recommendations are given based on the findings of the study. Professional nurses have expressed concerns about the shortage of staff. Staff shortage contributes to long patient waiting times, influx of chronic patients and additional workload, while also hindering the ability of professional nurses to provide adequate time to each patient. In light of this, the hiring of additional staff is recommended. The existence of inadequate infrastructure was identified as an additional major challenge that compromises the successful implementation of the ICDM model. Poor infrastructure affects the implementation of the ICDM model guidelines; therefore, it is recommended to address the issue of infrastructure in order to ensure the provision of comprehensive and integrated chronic disease management for patients.

The findings of the study indicate that adequate training is a key factor in the successful implementation of the ICDM model. Professional nurses’ training, hence, plays a crucial role in enabling them to effectively contribute to the success of the ICDM model, leading to improved patient outcomes in chronic disease management. It is, therefore, recommended that the authorities provide ongoing training and learning opportunities for professional nurses.

The study involved a small number of participants. Future studies on the subject, in other South African districts, are recommended so that those findings could subsequently be compared with those of the current study. Researchers should consider using larger sample sizes to build upon the findings of this study. Exploring the varying perspectives of all ICDM model users, including chronic disease patients and other healthcare professionals such as pharmacists and doctors, are other potential areas of study.

## Conclusion

As supported by previous studies, professional nurses in this study expressed positive perspectives on the perceived benefits of the ICDM model implementation such as – integrated care, adequate medication supply, improved efficiency and alleviation of stigma relating to diseases. This study, however, indicated that the ICDM model implementation is still being threatened by a variety of challenges, including – shortage of staff, poor infrastructure (for instance, small waiting rooms and few consultation rooms) and patients missing scheduled appointments.

In light of these challenges, the findings have emphasised the need for adequate staff, adequate infrastructure and ongoing training to enhance professional nurses’ performance within the ICDM model. This will promote their complete commitment to patient-centred care, to acquire knowledge or information about the latest advancements and evidence-based practices, in order to deliver the most effective and feasible care for patients who are managing chronic diseases.
